# Revealing mechanisms underlying variation in malaria virulence: effective propagation and host control of uninfected red blood cell supply

**DOI:** 10.1098/rsif.2012.0340

**Published:** 2012-06-20

**Authors:** C. J. E. Metcalf, G. H. Long, N. Mideo, J. D. Forester, O. N. Bjørnstad, A. L. Graham

**Affiliations:** 1 Department of Zoology, Oxford University, Oxford, UK; 2 MRC Epidemiology Unit, Institute of Metabolic Science, Addenbrooke's Hospital, Cambridge CB2 0QQ, UK; 3 Center for Infectious Disease Dynamics, Pennsylvania State University, Pennsylvania, PA 16802, USA; 4 Department of Fisheries, Wildlife, and Conservation Biology, University of Minnesota, MN 55108, USA; 5 Department of Ecology and Evolutionary Biology, Princeton University, Princeton, NJ 08544, USA

**Keywords:** malaria, evolution, health

## Abstract

Malaria parasite clones with the highest transmission rates to mosquitoes also tend to induce the most severe fitness consequences (or virulence) in mammals. This is in accord with expectations from the virulence–transmission trade-off hypothesis. However, the mechanisms underlying how different clones cause virulence are not well understood. Here, using data from eight murine malaria clones, we apply recently developed statistical methods to infer differences in clone characteristics, including induction of differing host-mediated changes in red blood cell (RBC) supply. Our results indicate that the within-host mechanisms underlying similar levels of virulence are variable and that killing of uninfected RBCs by immune effectors and/or retention of RBCs in the spleen may ultimately reduce virulence. Furthermore, the correlation between clone virulence and the degree of host-induced mortality of uninfected RBCs indicates that hosts increasingly restrict their RBC supply with increasing intrinsic virulence of the clone with which they are infected. Our results demonstrate a role for self-harm in self-defence for hosts and highlight the diversity and modes of virulence of malaria.

## 1. Introduction

Most pathogens exhibit broad genotypic variation in the extent to which they harm the host. Across a broad array of pathogen species (bacteria, viruses and protozoa), some clones within the species barely affect hosts at all, while others cause significant health impacts [1–4]. In murine malaria, the variation spans the range from mild disease that is quickly cleared by the host to severe disease that can kill the host [5]. Understanding what shapes this variation in virulence, both in terms of the biomedical mechanistic (proximate) and the evolutionary (ultimate) determinants, is of great public health interest [6]. For example, understanding the causes of varied virulence can help improve management of the immediate health consequences of infections caused by different clones and may also improve long-term prospects for controlling the evolution of virulence [7]. A glossary of terms appears in table 1.

**Table 1.** Glossary of terms.

term	definition
bystander killing	destruction of uninfected red blood cells by the immune system; uninfected cells can also be removed from circulation by the spleen
clone	genetically uniform lineage of malaria parasites
erythropoeisis	production of red blood cells
immunopathology	destruction of uninfected host tissue by the immune system
immune effectors	cells such as macrophages or proteins such as antibodies that are capable of damaging both parasites and host tissue
transmission	spread of a pathogen from one host to another or one cell to another
virulence	degree of harm to the host caused by infection; this includes direct damage by parasites as well as immunopathology

The standard evolutionary perspective on parasite evolution is that variation in virulence across clones should be shaped by the costs and benefits of virulence for parasite transmission [8–10]. The spread and persistence of virulence mutations may be favoured if they covary with transmission of the mutation. However, since host mortality can interrupt transmission, a range of virulence traits may lead to equivalent levels of overall fitness for the parasite [11]. While it is broadly agreed that the theoretical framework and empirical evidence on the existence of virulence–transmission trade-offs need to be further developed and extended [10,12], there are some systems where the general predictions appear to be borne out [13–15]. In particular, there is considerable evidence linking transmission rates to variation in the virulence of murine and human malaria clones [9]. In mice, malaria parasites that replicate more also produce more transmission stages, and cause more severe weight loss and anaemia of the host.

However, despite good evidence that malaria clones vary in intrinsic virulence [9], it is not yet clear whether virulence is achieved in the same way or via different mechanisms across clones. For the former, for example, all clones might be identical except in terms of the rate at which parasites invade uninfected red blood cells (RBC), and this rate would determine virulence. For the latter, variation might be found in several different characteristics of the parasite life cycle that would differentially impact virulence. This distinction has interesting implications both in terms of considering the impact of treatment and control measures (drug treatment, vaccination) within and across clones, but also from an ecological and evolutionary perspective. The degree of similarity in how parasites are exploiting host resources and evading immunity will shape the outcome of competitive interactions between clones [16,17], as well as the potential for clone coexistence. For example, some malaria parasite clones have been shown to have a high specificity for particular RBC age classes [2], and clones that target the same resources (e.g. reticulocytes (young RBCs)) may compete more strongly and be more likely to exclude each other than clones that show no overlap in resource use; the latter may be more likely to facilitate each other [18].

There is a further, important layer of complexity. Immunopathology has long been recognized in biomedicine as a key determinant of the degree to which host health is affected by parasites, and evolutionary biologists are increasingly inclined to agree [10,19–23]. Indeed, many of the negative health outcomes that we think of as malaria parasite virulence are mediated by the hosts’ own immune systems [23,24]. For example, cerebral malaria represents the combined pathogenesis of parasites and inflammation [24,25]. Furthermore, the immune system destroys (or removes from circulation) a substantial number of uninfected RBCs during malaria infection [26,27]. However, the destruction of uninfected RBCs may actually be contributing to the long-term control of the infection [28,29]. Establishing whether processes perceived as immunopathology (e.g. destruction of uninfected host tissue by the immune system) are primarily a component of virulence (e.g. collateral damage of parasite replication strategies) or primarily a component of defence (e.g. host strategies for limiting parasite resources) is critical to our understanding of host–parasite co-evolution.

Recently, statistical methods have been developed to partition causes of within-host malaria dynamics based on regression techniques of current parasitaemia on past infected and uninfected RBCs [29] that use measures of uninfected and infected RBCs one time-step in the past to partition mechanisms determining the present. This method enables the study of time-varying aspects of both host and parasite contributions to parasite dynamics. The methods centre around estimation of a time-varying quantity analogous to the transmission rate in canonical susceptible–infected–recovered epidemiological models, known as the effective propagation number, *P*_e_. Knowledge of *P*_e_ allows direct estimation of the time-varying within-host effective reproduction ratio *R*_e_ [30], or the average number of new infected cells per infected cell in a previously infected bloodstream. This is a key explanatory variable, because *R*_e_ > 1 indicates that the parasite population is growing, whereas *R*_e_ < 1 indicates that it is shrinking. Temporal fluctuations in both the effective propagation *P*_e_ and *R*_e_ may also reveal changes in the relative importance of different control mechanisms through time [29]. For example, in an experiment where mice were inoculated with different parasite densities [31], differences in effective propagation around the timing of the peak of parasitaemia indicate that adaptive immunity is delayed in hosts inoculated with lower parasite doses [29]. Such regression-based approaches also allow inference of time variation in host regulation of erythropoiesis and clearance of uninfected RBCs from circulation by immune effectors and the spleen.

Here, we use these new analytical techniques to explore the four following questions: (i) What are the similarities and differences among rodent malaria clones in the magnitude and time variation of effective propagation and net reproductive number?; (ii) What do these patterns reveal in terms of similarity and difference of underlying virulence mechanisms across clones?; (iii) Does the extent of immunopathology vary among clones?; and (iv) Can bystander killing, defined as the killing or removal of uninfected RBCs by the host during an infection, act as a host defence mechanism that ultimately aids control of parasites?

## 2. Material and methods

### 2.1. System and experimental set-up

**Figure 1.**
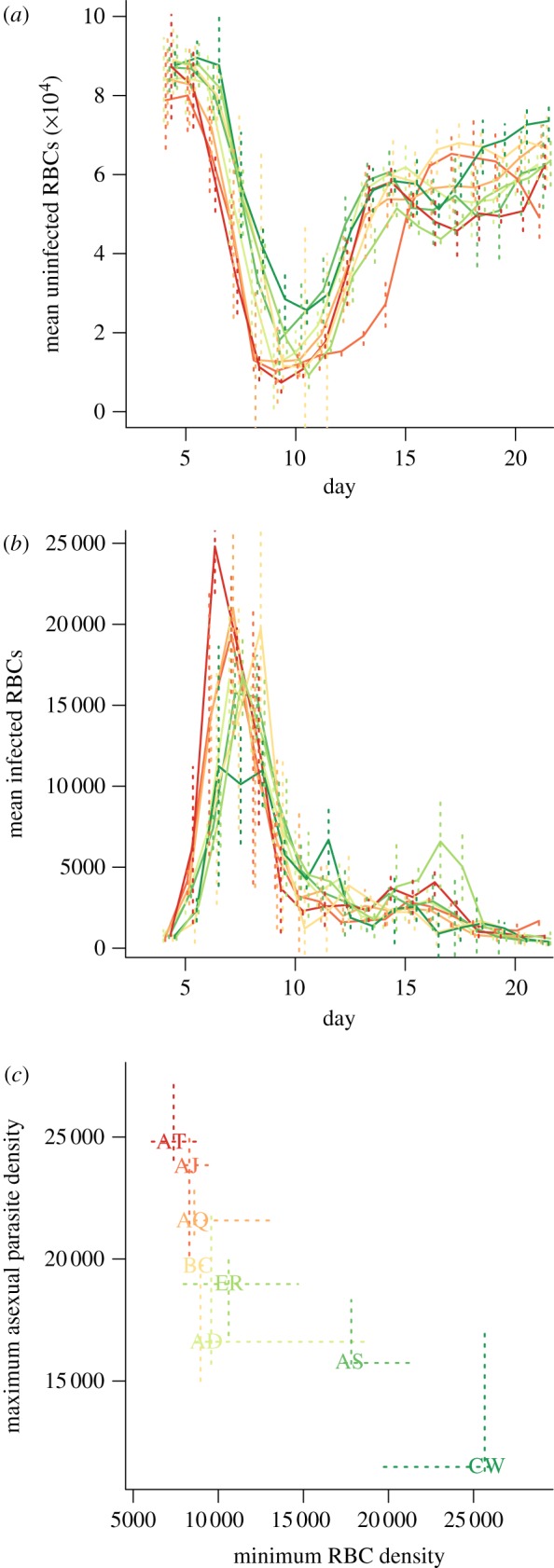
Time series of (*a*) mean uninfected and (*b*) infected RBCs per clone (×10^−2^ μl^–1^); vertical lines show standard deviations across five mice; and (*c*) relationship between maximum parasitaemia and depth of the RBC trough (×10^−2^ μl^–1^), suggesting the virulence–transmission trade-off, with dashed lines to indicate quartiles across individuals. Clone colours on the last panel correspond to those on the first two, and range from red for the clone resulting in the deepest RBC trough (AT), through to deep green for the clone resulting in the shallowest RBC trough (CW).

Laboratory mice frequently serve as model hosts for *Plasmodium chabaudi chabaudi* clones [25]. Every 24 h, and usually around midnight, infected RBCs burst, and the resulting merozoites must locate an uninfected RBC within a brief time span. For this experiment, eight genetically distinct *P. chabaudi chabaudi* clones were selected (AD, AJ, AQ, AS, AT, BC, CW, ER) based on the range of virulence they induce [32]. Infections were initiated with an injection of 10^6^ infected RBCs into five C57BL/6 female mice for each clone. RBC density and proportion infected (from which density of infected RBCs can be inferred) were measured daily from the fourth to the 20th day post-infection. Details are provided in previous descriptions of these experiments [33,34]. Figure 1 shows time-series of uninfected and infected RBCs for each clone, and the relationship between maximum asexual parasite density and minimum uninfected RBC numbers. Assuming that asexual densities are a good proxy for the density of the sexual transmission stages [9], this negative relationship indicates that clones that induce the highest maximum asexual parasite density also induce the most severe anaemia. These relationships are consistent and maintained across clones [33]. As seen here (figure 1*c*), and in previous work [9], the AS and CW clones show substantially less virulence than other clones in terms of weight and RBC loss. Materials and data are available upon request to C.J.E.M.

### 2.2. Parasite effective propagation

We estimate the effective propagation number, *P*_e_, a quantity analogous to the transmission coefficient in canonical models of between-host infection dynamics [29]. Temporal variation in *P*_e_ is shaped both by the effects of RBC age structure (predicted to shape parasite burst size, and invasion rates [35,36]) and immunity [35,37–40]. By analogy with between-host transmission ecology, given the discrete-time nature of malaria dynamics within the blood, if *I*_t_ is the number of infected RBCs at time *t*, and *S*_t_ is the number of uninfected RBCs at time *t*, then the expected number of infected RBCs observed at time *t* + 1 will be **λ**_t_ = *P*_e,t_ *S*_t_ *I*_t,_ where *P*_e,t_ is the effective propagation number of the infection at time *t*. This number can be thought of as the product of contact rates and transmission probability, given that a contact has occurred. Taking the log on both sides of the earlier-mentioned relationship, we can write
2.1
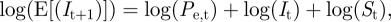

where E[(*I*_t+1_)] denotes the expectation. Applying regression techniques to (2.1) enables estimation of *P*_e,t_, whenever measurements of *I*_t_ and *S*_t_ are available. The within-host effective reproductive ratio is directly related to this quantity via *R*_e,t_ = *P*_e,t_ *S*_t_.

A subset of infected cells may in fact be committed to the production of transmission stages, and thus not contribute to *I*_t+1_, rather going on to produce gametocytes. However, we assumed for simplicity that the total observed infected cells could be equated with *I*_t_ in (2.1). Although cumulatively, the effect of this allocation towards sexual stages on the asexual densities may be substantial, on the one day time-scale considered here, the effect is likely to be very small, because the proportion committed is less than a few per cent relative to asexual stages [41].

### 2.3. Susceptible red blood cell dynamics

To also capture RBC replenishment and regulation, we can express an equivalent regression model for this element of the dynamics. Uninfected RBCs can be lost in three ways: via background mortality, estimated at around 0.025 *per capita* per day [42,43]; via immune mediated mortality (i.e. killing of uninfected RBCs by the immune system, or retention in the spleen, where they can neither carry oxygen for the host, nor provide resources for the parasite [26,27]; both of these are referred to here as ‘bystander killing’) and by becoming infected. Because we know numbers of infected RBCs at every time-point, we can explicitly account for this last process, and can write that the change in susceptible (uninfected) RBCs, denoted *S*_t_, is
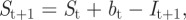

where *I*_t+1_ is total infected RBCs (encompassing both asexual infected RBCs, capable of continued within-host infection; and sexual infected RBCs, which cannot), and *b*_t_ is the total change in RBCs attributable to processes beyond the parasite (i.e. the balance of erythropoiesis (generation of new uninfected individuals, and mortality, as defined above). Because we have estimates for *S*_t_ and *I*_t_ at every time-step, *b*_t_ can be directly estimated. Values of *b*_t_ < 0 indicate that the loss of uninfected RBCs due to host-driven processes alone exceeds gains from erythropoiesis. Given background RBC mortality estimates of around 0.025 *per capita* per day [42,43] in the complete absence of erythropoiesis and taking the starting densities of RBCs (8 × 10^6^ μl^–1^, figure 1*a*), the loss of more than 20 uninfected RBCs per microlitre per day (8 × 10^4^ × 0.025 = 2000 × 10^−2^ μl^–1^) is unlikely without additional mortality via bystander killing. We therefore assume that values of *b*_t_ below −20 μl^–1^ d^–1^ indicate the occurrence of bystander killing.

**Figure 2.**
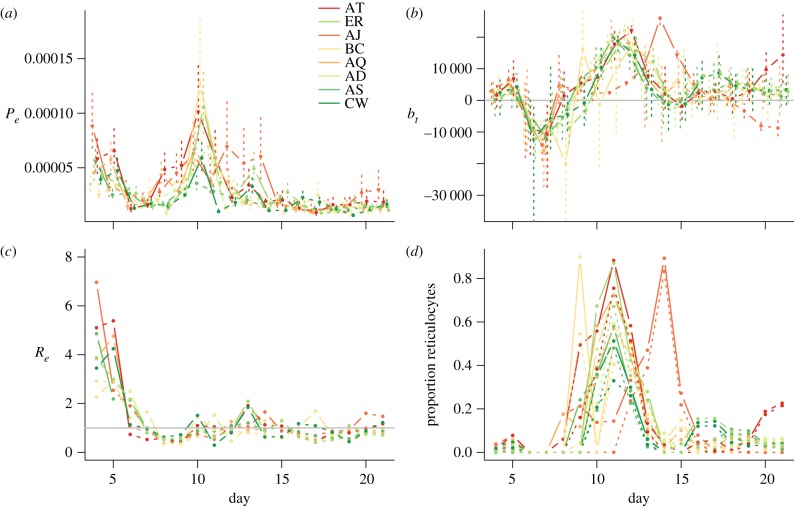
For eight clones (legend, colours reflect virulence measured as maximum anaemia), across days post infection (*x*-axis), (*a*) effective propagation *P*_e_ (standard errors shown as vertical lines); (*b*) changes in RBC density (×10^−2^ μl^–1^) not due to parasites (range across mice in each clone on each day indicated by vertical bars); values less than 0 (horizontal bar) indicate that destruction by immune effectors or the spleen exceeds replenishment; and (*c*) effective reproduction number, *R*_e_; values less than 1 (horizontal line) indicate that the parasite population is shrinking; and (*d*) proportion of reticulocytes at every time point obtained from combined RBC and parasite dynamics (see text); either assuming that normocytes are mostly infected (solid line) or assuming that reticulocytes are mostly infected (dotted line).

To fully resolve how RBC age structure affects susceptibility and transmission, studies that distinguish reticulocytes from normocytes, and further, to go beyond this to estimating the actual age of RBCs in days would be invaluable [39]. In the absence of that information, we can infer age structure via estimates of *b*_t_ using two boundary assumptions: that parasite attack rates are concentrated in either (i) the older, or (ii) the younger RBCs. Values of *b*_t_ > 0 will correspond to new RBCs entering into the population. Assuming first that infected cell mortality predominantly affects older RBCs (normocytes), the values of *b*_t_ > 0 will directly reflect the number of 1-day-old RBCs on day *t*, and the number of 2-day-olds the following day, etc. By contrast, for clones that preferentially invade younger RBCs, new RBCs entering the population on a given day (*b*_t_ > 0) are likely to be infected. Thus, to reconstruct the age structure, we subtract the number of parasites seen on that day from the estimate of *b*_t_ on that day, up to the limit of zero. If there are more parasites than the value of *b*_t_, the remainder can be subtracted from the 2-day-olds from the previous day, etc. Neither framework will provide an exact reflection of the age-structure, because preferences are unlikely to be so strict, and for young RBC preferring strains, there is no reason to assume 1-day-olds are targeted first. However, combining both assumptions of attack concentrated in either only young or only old RBCs, we obtain the full range of potential age structures. Given that our overall conclusions are not sensitive to either assumption (figure 2*d*), our study is robust to this uncertainty.

### 2.4. Implications of the host response for parasite dynamics

To explore the implications for every mouse of particular profiles of RBC replenishment and destruction as defined by *b*_t_, we can use the parametrized model to explore what would have happened had the observable uninfected RBC replenishment and depletion been modified (e.g. if hosts had been prevented from killing uninfected RBCs) by initiating a simulation with the number of infected and uninfected RBCs observed on the earliest day for which both measurements are available, and then simulating dynamics according to

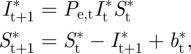



where *P*_e,t_ reflects the clone-specific effective propagation value for time-step *t* as estimated earlier, and *b*_t_* reflects values of *b*_t_ modified to the net effect of bystander killing of uninfected RBCs (by replacing negative values of *b*_t_ by zero).

The degree to which these simulations capture realistic within-mouse malaria dynamics hangs on the assumption that fluctuations in *P*_e,t_ and *b*_t_ are independent. If similar processes underlie their dynamics, altering *b*_t_ will result in alterations in *P*_e,t_ that the simulations will not reflect. However, given our limited knowledge of how the key processes play out dynamically, this approach allows us to at least explore the ‘what if’ scenario of changes in bystander killing, all else being equal.

## 3. Results

The overall time-pattern of *P*_e_ (figure 2*a*) broadly matches that found in a previous analysis focusing on the AS clone [29], with high early effective propagation that rapidly declines. However, many clones here also show a considerable increase in *P*_e_ close to the timing of the peak of parasitaemia (figure 2*a*, around day 10). This is correlated in time with a shift in the age structure of RBCs present in the blood towards reticulocytes (figure 2*d*). The subsequent decline in *P*_e_ after this peak (figure 2*a*) is likely to reflect engagement of adaptive immunity. The effective reproductive ratio R_e_ (figure 2*c*) shows similar patterns, although the increase around day 10 is considerably less marked because at this point mice are generally anaemic (figure 1*a*).

**Figure 3.**
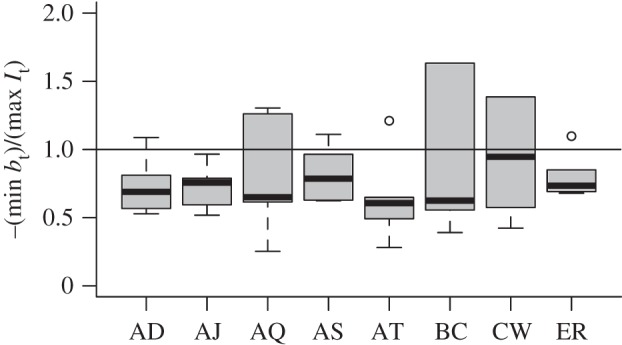
The ratio between minimum *b*_t_ and maximum *I*_t_ for mice within the eight clones. Values greater than 1 (horizontal line) indicate that more cells were killed by immunity or retention in the spleen in one time-step than by the parasite in one time-step, comparing the maximum of each for each mouse. The degree to which this occurs varies across clones.

The pattern of uninfected RBC production and destruction (figure 2*b*) is also similar to that seen in previous analysis [29] with RBC destruction or removal dominating for a period during the early exponential expansion of the parasite (*b*_t_ < 0 across all mice for each clone for at least one day before the peak of infection) followed by an increase in RBC production (*b*_t_ > 0 from around day nine, presumably in response to anaemia) and then generally a return to equilibrium as the infection is controlled. Despite these broad similarities, all clones show variation in timing and magnitude of patterns of *b*_t_, especially after day seven, and the response to clones AJ and BC deviate quite considerably from the others. The differences are significant: likelihood ratio tests for models of *b*_t_, including a random effect to capture mouse to mouse variation, and accounting for temporal autocorrelation (fitted with *lme* in the *nlme* package in R) reveal a highly significant interaction between ‘day’ and ‘clone’ (likelihood ratio test: *χ*^2^ = 2410, d.f. = 119, *p* < 0.001). For some clones, as many uninfected cells may be lost in one time step to bystander killing as are lost to parasites at the maximum peak of parasitaemia (e.g. for BC and CW, max(−*b*_t_)/max(*I*_t_) > 1, figure 3).

**Figure 4.**
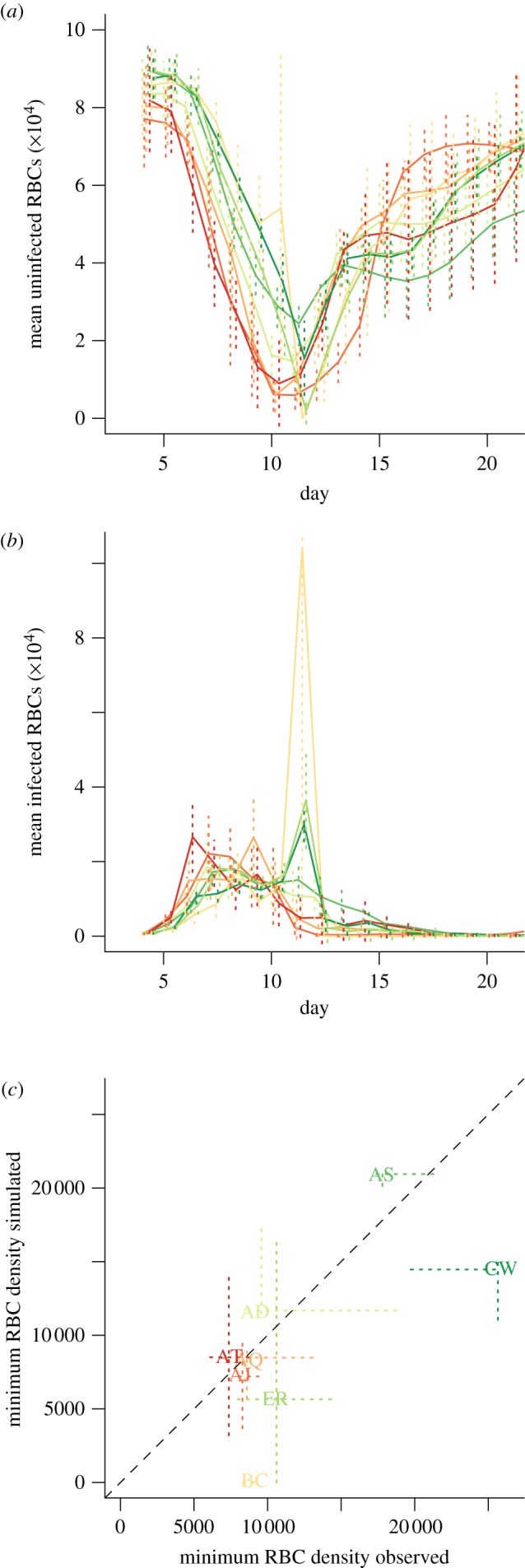
The effect of removing bystander killing or spleen retention on parasite virulence. (*a*) Simulated uninfected RBCs and (*b*) corresponding simulated infected RBC numbers (as in figure 1) obtained by taking individual-specific starting RBC densities and individual *b*_t_ values, and setting *b*_t_ to zero for time-steps when *b*_t_ < 0. (*c*) Observed minimum uninfected RBCs (×10^−2^ μl^–1^, *x*-axis) versus simulated minimum uninfected RBC obtained as described earlier (*y*-axis); vertical and horizontal lines indicate quartiles across individuals; the trough is deeper when no bystander killing is implemented for mice infected by several of the clones; exceptions include many mice in the AD and AS clones.

**Figure 5.**
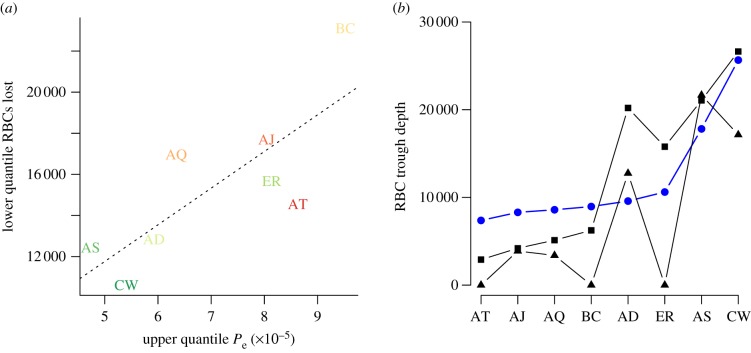
Strain differences in patterns of bystander killing and their impact (*a*) upper quantile of *P*_e_ for each clone, versus lower quantile of bystander killing (corresponding to the greatest loss) across individuals for that clone (×10^−2^ μl^–1^, as in figure 2*b*), indicating that clones that have highest effective propagation also experience the greatest magnitude of bystander killing (*n* = 8, *y* = −2846 + 17 8346 255*x*, *p* < 0.05, *r*^2^ = 0.56); (*b*) observed (blue circles) and simulated minimum uninfected RBCs (×10^−2^ μl^–1^, *y*-axis) for each clone, taking median starting densities across all mice for each clone, and introducing either the median *b*_t_ observed across all mice at that time step (black squares), or the maximum between this median value and 0 (black triangles, *b*_t_ > 0). Removing bystander killing increases anaemia in all cases, except for the AS clone, with differential magnitude effects across different clones.

Simulations that do not include bystander killing (figure 4*a,b*) can result in increased virulence in most mice, as measured by the depth of the RBC trough (figure 4*c*), but this result in only seen consistently in three of the eight clones. This may be partly due to covariation between the magnitude of bystander killing and clone maximum effective propagation: the lower quantile of RBC loss across mice is minimal for clones with the maximum effective propagation (figure 5*a*), i.e. highest levels of bystander killing are associated with mice infected by clones with the highest maximum propagation numbers (table 1).

To facilitate comparison of the effects of bystander killing across clones controlling for individual variability and clone specific effects on bystander killing, we also ran simulations taking median values of *b*_t_ across all individuals and clones, using clone-specific time-varying *P*_e_ values, and initiating simulations with the observed median starting values of infected and uninfected RBCs calculated across all individuals for each clone (taking the first day post infection for which both were available, here day four). Comparing median simulations with the observed time series indicates that, under median values of *b*_t_, mice infected with virulent clones would experience deeper RBC troughs than observed, and mice infected with less virulent clones would experience shallower troughs than observed (figure 5*b*, blue lines and black lines with squares intersect). Since *b*_t_ is broadly inversely correlated with the magnitude of clone effective propagation (figures 2*b* and 5*a*), the median *b*_t_ will represent an increase relative to the observed *b*_t_ for virulent clones and a reduction for avirulent clones. This pattern indicates that upregulation of erythropoiesis (and/or reduction of bystander killing) may counterintuitively worsen mouse health via increased anaemia that is caused by parasite-mediated RBC lysis. Removing bystander killing from the simulations resulted in consistently worsened anaemia for all clones except AS (figure 5*b*, triangles relative to squares, note that AJ also shows only a very slight improvement with anaemia). Although there is variability in the magnitude of this effect, it highlights the way in which temporal differences in *P*_e_ alone affect the impact of bystander killing on host health.

## 4. Discussion

Our analysis returned two major conclusions. First, although bystander killing might initially be considered a component of parasite virulence (e.g. with different parasite clones triggering different degrees of indiscriminate killing of uninfected RBCs by the immune system [44]), our results show that in standardized simulations, bystander killing actually reduces the maximum degree of anaemia experienced by hosts in all but one clone and is thus likely to be a component of the hosts’ malaria control strategy. Second, clone-specific temporal patterns of effective propagation through time (figure 2*a*) indicate the existence of intrinsic differences in clone exploitation characteristics that are not clearly organized along the virulence continuum, and that potentially reflect some form of niche partitioning [17,35,36]. Interestingly, however, although day-to-day fluctuations do not show a consistent pattern among clones (with, e.g. all the most virulent peaking on day 10), the maxima do (figure 5*a*).

Differential niche partitioning in malaria parasites must rely on variation across three main characteristics of their life cycle: (i) clones may differ in their rates of uninfected RBC invasion and infected RBC burst size (which may also vary through time); (ii) clones may differ in the degree to which the age of RBCs affects these two processes; and (iii) clones may differ in the degree to which they elicit immune responses, as well as the type of immune response they elicit. Unfortunately, data are not available to allow us to directly estimate the effects of RBC age on effective propagation [29], because these will be confounded with the impact of immunity on parasites (e.g. effective propagation might be reduced by immune-mediated elimination of infected cells or merozoites, or by changes in the age-structure of RBCs towards RBCs with lower burst sizes or invasion rates; and these processes will be indistinguishable). However, we can dissect the temporal patterns of *P*_e_ and *b*_t_ to identify which components are likely to be implicated.

Even from the very earliest days post infection, when differences in immunity among hosts infected with different clones might be expected to be negligible, and the RBC age structure should be essentially dominated by older RBCs (because RBC daily mortality rates are less than 0.05 per cent [42,43]), the various clones show different magnitudes of *P*_e_ and subsequently *R*_e_. For example, BC, one of the most virulent clones (figure 1*a*), shows rather low effective propagation on the first day of estimation (figure 2*a*), significantly lower than AJ, another virulent clone. These early time-step differences may partly reflect cross-clone variation in invasion rate or burst size in normocytes [29] but cross-clone variation in the degree to which immune responses are elicited (or, conversely, evaded) is likely to be the key; especially, given evidence that for doses of a comparable magnitude to those introduced here, both innate and adaptive immunity are active during the fourth–fifth day [29]. Previous work suggests that BC generally induces a high innate response relative to other clones (at least AJ, AS and CW), as measured by interferon (IFN)γ and tumour necrosis factor (TNFα) [33], lending further weight to the possibility that early differences in *P*_e_ are due to differential immune response induction.

The large peak in effective propagation occurring around day 10 for many clones is a surprising feature. It might be due to changes in the immunological context of the infection with a dip in immune efficacy around day 10, mirroring the double peak of immune efficacy used in other models [40]. If this is not the case, the pattern suggests a role for differential parasite responses to differently aged RBCs [36,40], either in terms of numbers of merozoites produced after an infected RBC bursts, or rates of uninfected RBC invasion. The proportion of reticulocytes in the blood stream around day 10 is likely to be high (figure 2*d*) because new RBCs will be entering the bloodstream as a result of considerably increased erythropoiesis for all clones around this time (figure 2*b*, *b*_t_ > 0 across clones for days after day 10). The fact that the peak in *P*_e_ is timed around day 10 suggests either higher burst size in reticulocytes, as supported by some experimental data [40], or higher invasion rates into reticulocytes. The opposite was suggested by previous models [36], but not borne out by subsequent experiments, which found them to be about equal [40]. The third possibility is that a combination of RBC age effects and waning of immune efficacy [40] is responsible for the observed peak in *P*_e_.

The importance of age preferences in determining malaria clone virulence has been highlighted previously [9]. For example, a single mutation that expands the age range attacked by *Plasmodium berghei yoeli* shifts the species from being a mild to a hypervirulent infection [45]. However, here our results suggest that links between the targeted age range and degree of virulence may be rather complex, because many of the avirulent clones as well as the virulent clones show an increase in effective propagation at around the time that the age structure is expected to transfer to being predominantly composed of young RBCs (figure 2*d*, day 10). By contrast, AS and AJ, which show less sensitivity to this transition, and therefore might be expected to have lower effective propagation (via invasion rate or burst size) in young RBCs are at opposite ends of the virulence spectrum.

Although complex immune patterns that covary with RBC age structure cannot be rejected [39,40], patterns throughout the time-series are consistent with persistent RBC age effects through time, and of relevance to overall virulence phenotypes. Clones that have highest *P*_e_ in older RBCs (e.g. AS, AJ, as evidenced by high early propagation, and a reduced response to the influx of reticulocytes around day 10, figure 2), reap the benefits of extremely high early *R*_e_, with AJ in fact producing a number of new infected RBCs close to the burst size (merozoite number per infected cell) reported for this clone [46]. By contrast, clones that apparently favour reproduction in younger RBCs do not experience large *R*_e_ values at the time-point corresponding to their largest *P*_e_, presumably because many other factors are in play, such as low RBC densities and strong immunity. Again, this does not translate into the virulence profile in any straightforward way.

In the face of this diversity of clone dynamics (figure 2*a*), it is of interest to ask whether the host should be employing a single generic defence strategy, or a variety of clone-specific strategies. We do not have direct information on how host immune systems are negotiating this challenge in terms of killing of infected cells (e.g. via clone-specific immune killing surfaces as in [29]). However, direct inference of erythropoiesis and bystander killing rates is possible (figure 2*b*). As mentioned earlier, this would indicate that hosts employ a very similar approach across clones in which they reduce the availability of uninfected RBCs via bystander killing during the exponential growth phase of the parasite (figure 1*b*), and then increase erythropoiesis during peak anaemia before returning to equilibrium. Beyond this general consistency, however, there is also variability linked to clone identity. Clone characteristics are correlated with the magnitude of host response, such that the highest levels of bystander killing were identified in mice infected by clones with highest rates of parasite growth (figure 5*a*). This specificity of the host's erythropoiesis and bystander killing response to different clones is also borne out by predictable changes in trough depth from simulations of ‘the median mouse’ for each clone, with either the same *b*_t_ imposed on all clones for each time-step (set to the time-varying median across the entire dataset), or the clone-specific median *b*_t_ (figure 5*b*). More virulent clones had shallower troughs in simulations using the across-clone median *b*_t_ value, as this was larger than their clone-specific *b*_t_ (figure 2*b*); less virulent clones experience the opposite (figure 5*b*).

Overall, these patterns suggest that the best strategy from the point of view of the host is to downregulate erythropoiesis (and/or engage in bystander killing) in proportion to the magnitude of RBC loss to parasitaemia early on. A proximate cue for such a strategy may arise from the production of TNFα by cells of the innate immune system upon binding malaria waste products as well as intracellular debris [47,48]. The data resolution available does not allow us to address this in detail, but we might expect reticulocyte-preferring parasite clones to be particularly vulnerable to reduction of erythropoiesis [28], while normocyte-preferring clones should be more vulnerable to bystander killing; an interesting avenue for future research.

While our analysis goes beyond early investigations that suggested that common mechanisms were regulating parasite growth across clones at all phases of the infection [9], there are still a number of key questions. In particular, our alteration of *R*_e_ by setting bystander killing to zero ignores many subtleties of RBC supply and regulation as well as the potential role of age effects. Time-points where *b*_t_ > 0 may also exhibit bystander killing or clearance from circulation by the spleen, but this signal may be swamped by the degree of erythropoiesis, which will also shift the age structure markedly towards younger RBCs, with potential implications for clones' effective propagation. We also assume that mortality of infected RBCs between the time of RBC invasion and the census time in the early morning is negligible. This is likely to be a robust assumption, because early during the cycle, infected RBCs are antigenically and structurally similar to uninfected RBCs. It is only over the course of their development that changes in membrane topology and display of antigens may occur; however, further empirical assessment of this and other assumptions will be valuable.

To conclude, for malaria, the virulence–transmission relationship is, on the face of it, rather simple—one uninfected RBC is lost every time a parasitized RBC is gained; so a negative correlation between peak parasite density and peak anaemia is to be expected in immune competent hosts. However, our analysis indicates that the underlying mechanisms and the way in which different clones line up along this continuum are actually rather complex. Effective propagation varies in magnitude across clones, and is higher at different times for different clones, owing to differential RBC age effects and immune killing of infected cells. Furthermore, host responses to different malaria clones differ in terms of RBC depletion (figure 5*a*), suggesting that clone-specific responses to parasites by the host can play a complex role in contributing to short-term measures of virulence (i.e. by increasing early anaemia) but thereby mediating the overall degree of virulence (including cumulative anaemia) by reducing *R*_e_ (figure 4). While dynamical analysis enables us to extend early work that focused solely on the timing and magnitude of peak of parasitaemia [49–51], a key direction for future work is to link the broad patterns we have described here to mechanistic differences among clones that account for their different immunological vulnerabilities and incorporate details on how they exploit specific RBC age structures and the host's clone-specific responses. Experiments incorporating a broad range of parasite genetic variation in inducing immunological responses will be a key part of this endeavour [52], as will reagents developed to manipulate the immune responses of laboratory mice. Finally, our results highlight that a complete understanding of natural selection on immunopathology [20] will require consideration of the role of immune-mediated killing of uninfected tissue in host defence.
